# Females and Their Diseases; a Series of Letters to His Class

**Published:** 1848-01

**Authors:** 


*Females and their Diseases; a Series of Letters to his Class.* By CHARLES D. MEIGS, M. D., Professor of Midwifery and the Diseases of Women and Children in the Jefferson Medical College of Philadelphia, etc. etc. etc. 8vo. pp. 670. Lea & Blanchard. Philadelphia: 1848.

This is the most *original* work that we have had occasion to notice for a long time. The author appears to think that medical subjects, except " the Physiology of Man," are " tiresome and disgusting," or, at least, wearisomely dull and uninteresting, when discussed in the plain didactic language generally employed by medical authors, and therefore he says, " I have endeavoured, according to my poor ability, to tell the truth about our Science and Art, and in doing so, to avoid the dulness—and I have thought the honestest way for a man to speak is, to speak what he thinks, in his own tone and manner, and not to come before the public under a false disguise."

A curious feature in the work is, the ever varying style. Although in the form of letters, we do not discover much of the epistolary character. At one time, we have the stateliness of a formal lecture; presently, it is a conversation with his pupils; and anon, a dialogue betwixt him and a lady patient! Some of the latter are droll beyond description, and but for the lack of time and space, we should be tempted to extract some specimens for the amusement of our readers. In one of these dialogues, with Miss Helen Blanque, a curious doctrine is unfolded, to which the author evidently attaches great importance, for we find the thread of it running through much of what is taught in the book. It concerns the lining membrane of the vessels—the *endangium*—or, as he calls it, the "*hæmatosic membrane.*" Addressing Miss Helen on the subject of the blood, he remarks:

" But all this blood is contained in those arteries, capillaries, or veins, we were just now speaking of. And the blood is not in contact with any part of your whole volume, save that inner tube, (endangium,) that inner quill within the swan quill; but, that inner tube is the ' common membrane of the blood-vessels,' and it would be much more sensible of us, if we would never call it by any other name than the BLOOD-MEMBRANE, or the blood-making membrane: and if I were not too well bred to speak Greek in your ears—a sort of 'leprous distilment'—I might call it the Hæmatosic membrane, a Greek word, that signifies blood-preparing, or blood-manufacturing membrane.

"If all the blood in your body touches this membrane, this tissue, and nothing else, then you have wit enough to perceive that, whatever be the cause of the production of the blood, that cause must exist in this hæmatosic membrane; I say, exist in it, either originally resident therein, or at least transmitted therethrough. This membrane is the ultimate, or the penultimate tissue in the hæmatosis.

"Suppose that membrane to be all right—all well—perfectly healthy and active in the performance of its duty in making 'the blood thereof'—don't you see that the 'life thereof' will be good and strong, and durable and pleasant? But, suppose, on the other hand, that the hæmatosic membrane is pale, flaccid, sick, weakly, good for nothing, can't you see that 'the blood thereof' will be like 'the life thereof'—good for little, or good for nothing?"

The idea, that the *endangium* or lining membrane of the vessels *manufactures the blood*, is new to us, and probably will be to the profession. What evidence is there of it? The author has adduced none, and we can conceive of none. Because all the blood in the body "touches this membrane," therefore the membrane must *manufacture* the blood, is a *non sequitur;* nor is it more logical to conclude, that the lining membrane of the blood-vessels is in fault in all modifications or derangements of the blood itself. When a woman floods largely, in a few hours it will be found that the absorbents have replenished the vessels; but the fluid will be poor and watery, and nearly unfit for the purposes of life. Now, what has the 'endangium' to do with this altered condition? Various substances are absorbed, and may be detected in the blood very speedily after being taken into the stomach, as iodine, turpentine, and the alkalies, &c. Here the blood is substantially changed in its condition, without the least instrumentality of "the hæmatosic membrane," as our author calls it. The blood, to a certain extent, is rendered either more or less alkaline, more or less crimson, and more or less consistent or fibrinous, according to the quality of the food from which it is made, and without the least reason for believing that any change has occurred in the "blood preparing membrane," any more than in every other part of the system.

The author's friendship, or exuberance of feeling, has in several instances led him into, what we conceive to be, extravagant eulogy of individuals, and occasionally into—what we are sure he does not intend—invidious comparisons. An instance of this occurs at page 566, where he speaks of Dr. Robert Lee as "the ablest man in all England, in this department (Midwifery) of science and practice." "I told him," says our author, "in 1844, that he might safely die now." "Why die?" said he. "Because you are already ineffaceably inscribed on the roll of Fame." "How's that? how's that?" said he. "It is thus, my dear doctor:—first, you are the man who made the profession know the true nature of phlegmasia-alba-dolens; it is you who have taught us the malady is crural phlebitis:—secondly, you are indissolubly united with that company of men who have established the spontaneous ovulation as a cause of menstruation. Nothing can separate your fame from that of Négrier, Gendrin, Pouchet, Purkinje, Von Baer, Rudolph Wagner, &c. Thirdly, you have just now shown me your last and greatest discovery, that of the largest ganglion in the human body, to wit, the cervical ganglion of the womb. I don't see any thing left for you to discover, and therefore, I say, you may die now; nothing can prevent you from being immortal." This broad compliment seems to have been received with the utmost complacency by the object of it; for he rejoined, "I'll no die yet! I'll no die yet!"

Apart from mere difference in taste, we confess we cannot agree with the author in this overstrained praise. We do not believe it is just, either to Dr. Lee, or to other parties whose claims conflict with his in the very matters indicated in the author's laudation; and if Dr. L.'s title to immortality is to rest upon the grounds he has placed it, we have great fears for its validity. He certainly was far from being the first who established that phlegmasia dolens is crural phlebitis. Dr. Lee himself states expressly, that "it does not admit of dispute, that Dr. Davis *was the first who proved by dissection that phlegmasia dolens depended on inflammation of the iliac and femoral veins.*" He himself claims the credit of having traced the inflammation to the uterine veins,—which cannot, of course, apply to crural phlebitis in man,—a claim which, after all, is a straining after originality not to be admired; and which may well be disputed, as other pathologists by no means accord, nor can they accord with the idea, that crural phlebitis necessarily begins in the uterine veins. "It is a remarkable circumstance," says Dr. Lee, "in the history of crural phlebitis, that nearly a century and a half should have elapsed from the time when it was first clearly pointed out by Mauriceau, before an opportunity was presented of ascertaining by dissection the precise nature of the disease. There had indeed been opportunities to determine the accuracy of the different hypotheses which had been advanced, but these were neglected, *and the seat of the disease and its commencement in the uterus were imperfectly understood* until the writer of this article" [Dr. Lee] "ascertained by dissection the true nature and origin of the complaint." This, be it observed, was in 1829, six years after the date which he himself properly assigns to the publication of Dr. Davis.

The other titles to immortality we esteem to be even more equivocal. In regard to Dr. Lee's views on the connection between ovulation and menstruation, he is only one of several who maintained them; and as to his description of the nerves of the uterus, although there are many who place credit in them, the Royal Society of London, by approving of, and crowning, the investigations of Mr. Beck—has virtually expressed the belief, that many of his so-called nerves have no title to be regarded as such. We may do Dr. Lee injustice—we are far from wishing to do so—when we state, that his course, in regard to reputed discoveries, and to his eminent fellow-observers, has not appeared to be magnanimous, or even in all cases characterized by over-liberality and accuracy. In the controversy with Mr. Beck, Dr. Sharpey and others, in regard to the uterine nerves, there certainly appeared to be too great a desire to elevate himself and his investigations at the expense of the fair fame of others; and we were pained to be compelled to arrive at this conclusion, and to have the respect which we had previously entertained for him, diminished by the merited castigations which his exacting statements in the journals drew down upon him. In the controversy, too, which occurred between him and Dr. R. Paterson, of Leith, on the subject of the corpus luteum, about three years ago, the characteristics of the *genus irritabile* were equally apparent; and, what was worse, faulty observation was too evidently associated with them. In the 142d number of the *Edinburgh Medical and Surgical Journal*, Dr. Paterson described what he conceived to be an early corpus luteum. This was designated by Dr. Lee, in his lectures, as a mere clot of blood; and he afterwards affirmed, that "the said clot of blood did not present one of the characters of a true corpus luteum," and that if he "was summoned into a court of justice, he would have no hesitation in declaring upon oath, from the evidence furnished, that the proofs of pregnancy were wholly wanting." Dissatisfied—as well he might be—with these remarks, Dr. Paterson forwarded, through a friend, the *identical specimen*, without stating whence it came, in order that Dr. Lee's unbiassed opinion of it, from personal inspection, might be obtained; when, after a careful examination, Dr. Lee returned an answer, *declaring it to be an early corpus luteum, and requesting permission to describe it as such before the Royal Medico-Chirurgical Society of London!!*

Even in one of the very last numbers of the London "Lancet" (for Nov. 6, 1847) which we have received, we find him again in strife; charged by Dr. Fleetwood Churchill—a respectable name—with having "most unwarrantably published," without his consent, a letter hastily written to him two years previously. "That," says Dr. Churchill, "was, to say the least of it, uncourteous; but to distort its admissions until the statement becomes untrue, and to employ my own "candour" and "frankness" to disqualify my entire calculations, is an offence of a much graver character." We have not space to enter into the subject of the precise cause of difference between Dr. Churchill and Dr. Lee; but no unprejudiced person can read the correspondence without arriving at the conclusion that the right seems to be altogether with the former.

In another number of the same journal, (Nov. 20, 1847,) we observe that Dr. Lee is also engaged in a controversy with Professor Simpson, of Edinburg, towards whom he employs language which is certainly unusual in discussions among elevated members of our profession.

The author ridicules the idea of the existence of such diseases as *physometra* and *hydrometra.* With reference to the first of these, he remarks: "I cannot patiently endure to think that a pupil of mine, be he settled in Maine or Wisconsin, at the Sault St. Marie or Monterey, should admit to a patient that the womb can become filled and distended with gas, as a result of diseased secretion, for such secretion is impossible, and to admit it is ridi culous."

We cannot avoid the expression of our regret, that such language as "impossible," and "ridiculous," should be employed by so high an authority in reference to any scientific opinion, and especially to a phenomenon, which is unhesitatingly admitted by all the distinguished pathologists of the day. Physometra, too, is admitted by most writers on the subject, and avouched by numerous observers, among whom may be mentioned Astruc, Frank, Gooch, and Ashwell.

To deny what we have not ourselves seen, because we have not seen it, and because it does not exactly square with our *à priori* reasoning, is not courteous; and, moreover, it is a dangerous precedent for the young and inexperienced—an example that should not be set hastily by those who desire that their own statements may be implicitly received.

The author takes the bold ground that air (we understand him to mean gaseous matter of any kind,) is never secreted;—"Did you ever hear of air being secreted by the bladder of urine? Never. Air is not secreted. The bubbles that you see upon the skin, are not bubbles of secreted air; they arise from the vapourization, or from the transformation of fluid products on the skin. A fish even does not secrete the air of his swimming bladder. He comes to the surface for it."

In this extract it seems to us that we discover several errors of fact. In the first place, we have a distinct recollection of at least one well attested case of air being discharged from the bladder mixed with the urine. In the second place, we would ask, whence come the bubbles of air seen over all the body when lying tranquilly in a bath of clear water?

Under the head of *Pneumatoses*, in his Pathological Anatomy, Philadelphia edition, Vogel has the following remarks:

"*Gases may be actually secreted by different parts of the body.*"

"Thus, Magendie and Girardin assert, that on confining a portion of the intestine of a live dog between two ligatures, in the course of some hours the included portion was found full of air, which escaped with a hissing sound on making an incision. In the intestinal canal of swine we sometimes meet with considerable accumulations of gas between the layers forming the walls of the bowels. Sir Francis Smith has described an interesting case of the development of gas in man, which deserves a full notice. He states: 'On the 12th of May, 1840, I was consulted by a gentleman, who told me that he often suffered from an enormous development of gas in the stomach, which he discharged by eructation; that he likewise, occasionally, experienced a development of gas from the bladder, and that his skin acted in a similar manner, as he had observed in the bath. On the morning of the 15th, I found my patient in a bath at 79° F. His breast, shoulders, abdomen, and hands, were literally covered with minute bubbles of gas. On being questioned, the attendant at the bath stated that he had never previously witnessed anything of the kind. On removing the hands and arms from the water, the air bubbles disappeared, but gradually returned on again immersing those organs. The bubbles were of the size of a pin's head. On wiping them off, they disappeared, but gradually formed again."

With regard to the swimming bladder of fishes, we know that it is ranked by naturalists as a part of the respiratory apparatus, but there are many fishes in which it can subserve no such purpose—where, at least, no communication whatever with the external air can be discovered, and where the air in it cannot be procured by the "fish coming to the surface for it."

But whence comes the prodigious quantity of gas that sometimes proceeds from the stomach and bowels? We lately had a lady patient from whose stomach gas issued during a great part of the day and night, in such quantity as to be audible not only in the room beneath where she was, but even in the street; and a professional friend of ours, during a severe illness, was obliged to keep a tube continually in the anus to allow of the escape of gas from the bowels for many days. In these cases it was not to be believed, from the quantity of gas discharged, that it could have proceeded from the decomposition of ingested matter; and besides, it was without any perceptible odour. By the way, Dr. Churchill lays it down as a rule that, "in idiopathic physometra, the gas is inodorous, but not so when the result of decomposition."

Our author is equally incredulous on the subject of hydrometra. "A bunch of hydatids is a *dropsical placenta*, and a dropsical placenta is the dropsy of the womb, or hydrometra, of which you have heard." In this view of the disease, the author may be right, although most writers regard hydatids and hydrometra as distinct diseases, and the autopsies reported by Dr. Thompson, and others, seem to justify this distinction.

There are many other subjects embraced in this interesting volume, of which we should be glad to say something, but already we have greatly exceeded the limits usually assigned to such notices. The work, as the author remarks, is made out of his own brains—the scissors have been but little employed. The first edition of such a work, and especially when coming from one of the most popular practitioners and teachers that the country has produced, demanded more than a passing notice, whilst truth, science, and a proper regard for the author, required that the notice, whether brief or extended, should be altogether frank. Our strictures, however, have occupied so much space as to exclude the commendations to which portions of the volume are entitled, and which it has been our desire to bestow. The reader nevertheless will have no difficulty in discovering the excellent counsel expressed on many of its pages, and having commenced their perusal, he will not be likely to desist until he has read every paragraph in the book. The style, as well as some of its doctrines, we are apt to think, will be sharply criticised. But as the author remarks, "Whether such a mode of writing may prove agreeable to the brethren, so as to meet their approbation, remains to be seen."

As might be expected in a first edition, driven through the press with all the rapidity of steam, as is the custom with Philadelphia publishers, some awkward typographical errors have escaped the proof-reader. These, (and, we hope, some of the points to which we have alluded,) will undergo the author's careful revision in a future edition, which, we predict, will be demanded at an early day.

